# A report on the physicochemical and antioxidant properties of three Indonesian forest honeys produced by *Apis dorsata*

**DOI:** 10.1016/j.fochx.2025.102156

**Published:** 2025-01-03

**Authors:** Mahani, Pamungkas Rizki Ferdian, Habil Muhammad Ghibran, Amirah Fathia Herlina, Siti Nurhasanah, Nunung Nurjanah, Rizki Rabeca Elfirta, Avry Pribadi, Raden Lia Rahadian Amalia, I Made Samudra

**Affiliations:** a Department of Food Industrial Technology, Faculty of Agro-Industrial Technology, Padjadjaran University, P.O. Box 45363, Sumedang, Indonesia; b Research Center for Applied Zoology, National Research and Innovation Agency Republic of Indonesia, P.O. Box 16911, Bogor, Indonesia; c Research Center for Public Health and Nutrition, National Research and Innovation Agency Republic of Indonesia, P.O. Box 16911, Bogor, Indonesia; d Research Center for Applied Microbiology, National Research and Innovation Agency Republic of Indonesia, P.O. Box 16911, Bogor, Indonesia

**Keywords:** Antioxidant, *Apis dorsata*, Forest honey, Indonesian honey, Physicochemical, tropical honey

## Abstract

Indonesia, one of the largest tropical forests, offers a diverse range of nectar sources that contribute to the unique characteristics of forest honey. This study aims to investigate physicochemical and antioxidant properties of *Apis dorsata* forest honey from three distinct regions of Indonesia. Key physicochemical parameters include moisture, color, electrical conductivity (EC), total dissolved solids (TDS), total suspended solids (TSS), density, diastase number (DN), hydroxymethylfurfural (HMF), pH, total acidity, ash content, protein content, and reducing sugars. Antioxidant properties, assessed through total phenolic content (TPC), total flavonoid content (TFC), DPPH radical scavenging activity, ascorbic acid equivalent antioxidant capacity (AEAC), and ferric reducing power (FRP), revealed significant regional variability. Principal component analysis (PCA) distinguished honey samples based on these attributes. These findings provide preliminary insights into the variability of Indonesian forest honeys. However, the small sample size limits generalizations, and further research with larger datasets is essential for validation.

## Highlights

• Indonesian tropical forest honey from *Apis dorsata* offers rich antioxidant compounds.
• Indonesian forest honey's physicochemical properties vary significantly across regions.
• Antioxidant activities differ among honey samples from Ternate, Siak, and Papua.
• Key physicochemical properties correlate strongly with antioxidant characteristics.
• Geographical origin and local floral sources impact honey's quality and antioxidant potential.

## 1 Introduction

The *Apis dorsata* bee is a wild bee species commonly found throughout Indonesia that cannot be cultivated. They play a significant role in honey production in the country, contributing an estimated 70–80 % of the total honey output. Honey bees depend on nectar and pollen for their survival, and Indonesia's diverse plant species offer a wide range of nectar sources, resulting in various types of honey (Evahelda, Pratama, Malahayati, & Santoso, 2017). Indonesia is home to approximately 115 plant species that serve as nectar sources for honey bees. The honey produced by *A. dorsata* bees, often referred to as multifloral or forest honey, originates from the diverse nectar sources in forested areas. This multifloral honey is not only influenced by the bee's environment but also by its complex composition. Honey contains over 200 compounds, including proteins, enzymes, vitamins, minerals, phenolic acids, antioxidants, and carbohydrates, with sugars comprising about 80 % of its content. Simple sugars like fructose and glucose are easily metabolized by the bee's digestive systems (Bogdanov et al., 2008). Among them, phenolic compounds play a significant role in the honey's biofunctional properties, particularly antioxidant activity.

Honey is a rich source of antioxidants and offers a variety of medical and health benefits. It has been reported to exhibit bioactivities such as antioxidant (Moniruzzaman et al., 2013; Saxena et al., 2010; Shamsudin et al., 2019), antiomicrobial (Mduda et al., 2023; Saputra & Nurlina, 2022; Ávila et al., 2019), anticancer (Ahmad et al., 2019), and anti-inflammatory (Borsato et al., 2014) properties, making it a promising therapeutic agent for preventing and treating various diseases, including cancer, cardiovascular diseases, inflammatory disorders, neurological degeneration and aging. Antioxidants in honey protect the body from damage caused by free radicals, thereby preventing various diseases. If the body's antioxidants are insufficient to counteract oxidation, free radials can attack cells, particularly lipids and proteins, leading to degenerative diseases associated with premature aging (Martemucci et al., 2022). While the body can produce endogenous antioxidants which act as protective compounds against free radicals, their production is limited. As a preventive measure against diseases caused by oxidative stress, it is essential to consume foods rich on antioxidants.

Exogenous antioxidants can be obtained either synthetically or naturally. Natural antioxidants are derived from foods made from natural ingredients and are considered safe with no adverse side effect on health. Honey is A natural food ingredient with significant antioxidant potential and is recognized as a functional food. It has been demonstrated to be a promising therapeutic agent for various diseases due to its antioxidant properties.

However, the biological content of honey and the total antioxidant capacity of honey are determined by the geographical and botanical origins as well as the type of bee species. Honey's composition and bioactive compounds largely depend on floral source, geographical location, seasonal variations, and environmental conditions (Martinello & Mutinelli, 2021). These factors not only influence honey's bioactive components but also result in diverse physicochemical properties. The physicochemical characteristics of honey are critical indicators of its quality.

This study, based on a limited sample size, aims to explore the physicochemical and antioxidant properties of Indonesian forest honey produced by *A. dorsata* as a basis for future, more comprehensive investigations.

## 2 Materials and methods

### 2.1 Materials

#### 2.1.1 Sample collection and handling

**Fig. 1:**
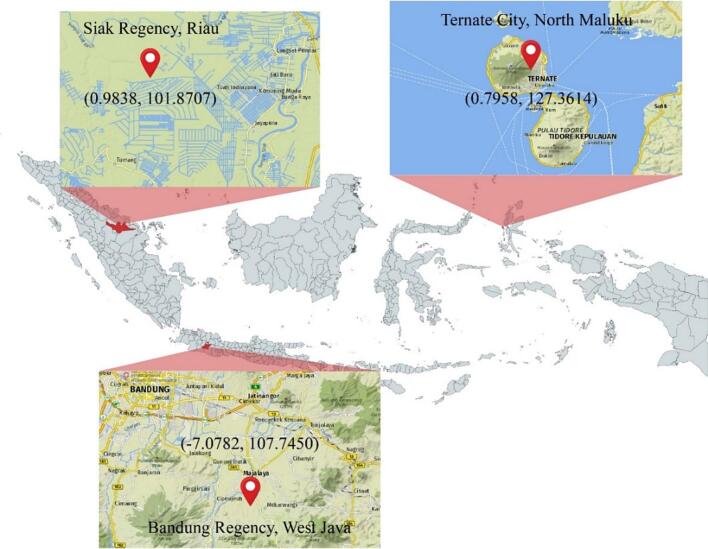
Indonesian *Apis dorsata* honey source regions.

Three freshly *A. dorsata* honey samples were sourced from different regions across Indonesia (Fig. 1) by local honey hunters. The samples were immediately transported to the National Research and Innovation Agency Republic of Indonesia in Bogor, West Java. Upon arrival, they were stored in an air-conditioned room maintained at 25 °C with minimal light exposure. The samples were then aliquoted into 50 mL tubes for analysis. Detailed information regarding the nectar sources, collection locations, and their corresponding geographical coordinates is provided in Table 1.

**Table 1:** Indonesian *Apis dorsata* honey nectar sources, origins, and coordinates.

Geographical Origin	Nectar source	Latitude, Longitude	Harvest Time
Siak Regency, Riau	Dominant accacia	0.9838, 101.8707	2022/2023
Ternate City, North Maluku	Multiflora	0.7958, 127.3614	2022/2023
Bandung Regency, West Java	Multiflora	−7.0782, 107.7450	2022/2023

#### 2.1.2 Chemicals and reagents

All chemicals and reagents used in present study were of pro-analytical grade. Acetic acid, aluminum chloride, ascorbic acid, bovine serum albumin (BSA), Coomassie Brilliant Blue G-250, 3,5-dinitrosalicylic acid (DNSA), ethanol, ferric chloride, Folin-Ciocalteu reagent, glucose, hydrochloric acid, iodine, methanol, phenol, phosphate buffer 0.2 M pH 6.6, potassium ferrocyanide, potassium iodide, potassium sodium tartrate, phosphoric acid, sodium acetate, sodium bisulfite, sodium carbonate, sodium chloride, sodium hydroxide, sodium metabisulfite, starch, trichloroacetic acid (TCA), zinc acetate were purchased from Merck. Quercetin, gallic acid, and 2,2-diphenyl-1-picrylhydrazyl (DPPH) were obtained from Sigma Aldrich.

### 2.2 Methods

#### 2.2.1 Moisture content (MC)

The moisture content (MC) of honey samples was determined according to Bogdanov et al. (1997) in triplicate. Using a portable refractometer, a few drops of honey were placed on the prism until fully covered. The MC was read directly through the refractometer display and expressed as percentage (%).

#### 2.2.2 Color analysis and intensity

Honey color was analyzed using a spectrophotometer (Konica Minolta, Model: CM-5, Osaka, Japan) following Saxena et al. (2010). The honey sample was positioned within the cuvette, and the Lab color space values (L*, a*, b*) were recorded. The L* value indicates the lightness, while a* and b* represents the red/green and yellow/blue color coordinate, respectively.

Color intensity (CI) was meassured following Moniruzzaman et al. (2013) with slight modifications. The honey sample was diluted in deionized water to create a 50 % (*w*/*v*) solution. After centrifugation to remove solids, the supernatant was collected, and its absorbance was measured at 450 nm and 720 nm using a UV–Vis spectrophotometer (Thermo Fisher Scientific Inc., Model: Genesys 10-S, Waltham, USA). Color intensity was calculated as the difference in absorbance at these wavelengths and expressed in mAU. Both color analysis and intensity were measured in triplicate.

#### 2.2.3 Electrical conductivity (EC), total dissolved solids (TDS), and total suspended solids (TSS)

The EC and TDS of honey samples were measured using an EC-TDS meter for a 20 % (*w*/*v*) honey solution, in triplicate (Khalil et al., 2012; Moniruzzaman et al., 2013). EC was expressed in mS/cm, while TDS was expressed in μg/mL (Bogdanov et al., 1997). To determine TSS, honey samples were diluted in hot distilled water (100 °C) to make a 10 % (*w/v*) solution. The solution was filtered using pre-weighed filter paper to separate insoluble solids. The filter paper with retained solids was dried at 105 °C for 2 h, cooled in a desiccator, and then re-weighed until a constant weight was achieved. TSS content was calculated and expressed as grams per 100 g of honey (Bogdanov et al., 1997). Analysis was conducted in triplicate.

#### 2.2.4 Density

Honey density was measured in triplicate by accurately weighing 1 mL of each sample in a pre-weighed microtube using analytical balance (Mettler Toledo, Model: AT261, Switzerland). The same volume of pure water, with a density of 1.0 g/mL, was weighed as a standard reference.

#### 2.2.5 Diastase number (DN)

Diastase activity was determined based on the Standar Nasional Indonesia, (2018). Five grams of honey were mixed with 10–15 mL of distilled water, 2.5 mL of acetate buffer, and 1.5 mL of sodium chloride solution. The mixture was diluted to 25 mL, and 10 mL of the solution was combined with 5 mL of starch solution. The sample mixture was incubated at 40 °C in a thermostatic bath. At 5-min intervals, 1 mL of the mixture was combined with 0.0007 N iodine solution, and absorbance at 660 nm was measured using a UV–Vis spectrophotometer (Thermo Fisher Scientific Inc., Model: Genesys 10-S, Waltham, USA). The time (t) required to reach an absorbance of 0.235 was calculated using a linear curve between absorbance and time intervals. DN was calculated in triplicate using the following equation:

$$\mathrm{Diastase number}\;\left(\mathrm{DN}\right)=\frac{300}{t}$$

#### 2.2.6 Hydroxymethylfurfural (HMF) content

The HMF content of honey was determined following the Standar Nasional Indonesia, (2018). Five grams of honey were dissolved in 25 mL of distilled water, followed by the addition of 0.5 mL each of Carrez I and Carrez II reagents, and diluted to 50 mL. The solution was filtered, and 5 mL of the filtrate was divided between two tubes- one as the sample solution and the other with 0.2 % sodium bisulfite added as referene. Absorbance readings were taken at 284 nm and 336 nm using a UV–Vis spectrophotometer (Thermo Fisher Scientific Inc., Model: Genesys 10-S, Waltham, USA). HMF content was was calculated based on absorbance differences and expressed as mg/kg using the following equation:

$$\mathrm{HMF}\;\mathrm{content}\left(\mathrm{mg}/\mathrm{kg}\right)=\frac{\left(\mathrm{Abs}\;284-\mathrm{Abs}\;336\right)x\;149.7\;x\;5\;x\;\mathrm{Dilution factor}}{\mathrm{Weight of sample}}$$

#### 2.2.7 Total acidity (TA) and pH value

Total acidity was determined in triplicate using potentiometric titration (BSN, 2018). For free acidity, 10 g of honey was diluted in 75 mL of CO_2_-free distilled water and titrated with 0.1 N NaOH until pH 8.50. Lactone content was measured by titrating 10 mL of 0.1 N NaOH with 0.1 N HCl, stopping at pH 8.30. Total acidity was calculated as the sum of free acidity and lactone values, and expressed as milliequivalents of NaOH per kilogram (meq NaOH/kg) of honey. Subsequently, pH was measured on a 10 % (*w*/*v*) honey solution using a pH meter (TOA Electronics Ltd., Model: 45CU223W, Japan) (Bogdanov et al., 1997).

#### 2.2.8 Ash content

Ash content was determined using the gravimetric method in triplicate. A precisely weighed 1 g of honey sample was placed in a pre-weighed porcelain crucible using analytical balance (Mettler Toledo, Model: AT261, Switzerland). The sample was incinerated at 600 °C for 5 h, cooled in a desiccator, and re-weighed. Ash content was expressed as grams of ash per 100 g of honey (AOAC, 1990).

#### 2.2.9 Protein content

Protein content was measured using the Bradford method (Azeredo et al., 2003). A 50 % (*w*/*v*) honey solution was prepared, and 0.1 mL aliquot of the sample solution was mixed with 5 mL of Bradford reagent. After a 10 min incubation, and absorbance was measured at 595 nm using a UV–Vis spectrophotometer (Thermo Fisher Scientific Inc., Model: Genesys 10-S, Waltham, USA). Protein content was calculated using a BSA standard curve and expressed as μg/mL.

#### 2.2.10 Reducing sugar content

The reducing sugar content of honey was determined using a DNSA method, as described by Moniruzzaman et al. (2013) with slight modifications. A 500 μg/mL honey sample solution was prepared in distilled water. A 0.5 mL aliquot of this solution was mixed with 1 mL of DNSA reagent (prepared by dissolving 3.50 g NaOH in 250 mL of distilled water, combined with 1.87 g of 3,5-dinitrosalicylic acid, 54.03 g of potassium sodium tartrate, 1.34 g of phenol, and 1.47 g of sodium metabisulphite). The mixture was heated in a boiling water bath for 5 min and then cooled to room temperature. Subsequently, 5 mL of distilled water was added, amd the absorbance was measured at 540 nm using a UV–Vis spectrophotometer (Thermo Fisher Scientific Inc., Model: Genesys 10-S, Waltham, USA). A glucose standard curve was used to quantify the reducing sugar content, which was expressed as grams of reducing sugars per 100 g of honey.

#### 2.2.11 Total phenolic content (TPC)

The TPC was determined using the Folin-Ciocalteu method. One gram of honey was dissolved in 10 mL of distilled water. Then, 0.2 mL of the sample was mixed with 1.8 mL of distilled water and 0.2 mL of Folin-Ciocalteu reagent. The mixture was homogenized and incubated for 6 min. Next, 2 mL of 7 % (*w*/*v*) sodium carbonate solution was added, and the solution was homogenized again. After a 90 min incubation, the absorbance was measured at 750 nm using a UV–Vis spectrophotometer (Thermo Fisher Scientific Inc., Model: Genesys 10-S, Waltham, USA). TPC was calculated and expressed as milligrams of gallic acid equivalents per kilogram of sample (mg GAE/kg sample). Analysis was conducted in triplicate.

#### 2.2.12 Total flavonoid content (TFC)

The TFC was determined by the Dowd method spectrophotometrically. One gram of honey was dissolved in 10 mL of distilled water. Then, 1 mL of the sample solution was mixed with 0.2 mL of a 10 % aluminum chloride solution, 0.2 mL of 1 M sodium acetic, and 5.6 mL of distilled water. The solution was incubated for 30 min, and absorbance was measured at 415 nm using a UV–Vis spectrophotometer (Thermo Fisher Scientific Inc., Model: Genesys 10-S, Waltham, USA). The TFC was expressed as milligrams of quercetin per kilogram of honey (mgQE/kg sample). Analysis was conducted in triplicate.

#### 2.2.13 Antioxidant activity by DPPH assay

Antioxidant scavenging activity was determined using the method described by Saxena et al. (2010). A total of 0.5 mL of the diluted honey sample was combined with 1.5 mL of 0.1 mM DPPH solution in methanol. The mixture was incubated in the dark at room temperature for 30 min, after which the absorbance was measured at 517 nm using a UV–Vis spectrophotometer (Thermo Fisher Scientific Inc., Model: Genesys 10-S, Waltham, USA). The DPPH free radical inhibitory activity was expressed as IC_50_. The percentage of DPPH radical inhibition at each sample concentration was calculated using the following formula:

$$\%\mathrm{inhibition}=\frac{\mathrm{Ablanko}-\mathrm{Asample}}{\mathrm{Ablanko}}\;x\;100\%$$

In addition, antioxidant capacity using DPPH assay was also evaluated. The DPPH radical inhibition at a single concentration was used to calculate the ascorbic acid equivalent antioxidant capacity (AEAC) (Moniruzzaman et al., 2013). This was determined using standard curve of ascorbic acid and expressed as milligrams AEAC per kilogram of sample (mg AEAC/kg sample).

#### 2.2.14 Antioxidant activity by FRP assay

The FRP assay was conducted to determine antioxidant activity by measuring the reduction of ferric ions, as described by Jafri et al. (2017). A 0.3 mL of the sample solution was combined with 0.6 mL of phosphate buffer (0.2 M; pH 6.6) and 0.6 mL of 1 % (b/v) potassium ferrocyanide. The mixture was incubated at 50 °C for 30 min. Afterward, 0.6 mL of 10 % TCA was added, and the solution was centrifuged at 3000 rpm for 10 min. Following centrifugation, 0.5 mL of the supernatant was mixed with 0.5 mL of distilled water and 0.1 mL of 0.1 % (*w*/*v*) ferric chloride. Absorbance was measured at a wavelength of 700 nm using a UV–Vis spectrophotometer (Thermo Fisher Scientific Inc., Model: Genesys 10-S, Waltham, USA). Standard curve of ascorbic acid was used to determine ascorbic acid equivalent (AAE) and expressed as milligram AAE per kilogram of honey (mg AAE/kg sample).

### 2.3 Statistical analysis

All experiments were conducted in triplicate, and the results were expressed as mean ± standard deviation. Significant differences between samples were analyzed using one-way analysis of variance (ANOVA), followed by Tukey's honestly significant difference (HSD) post hoc test (*p* < 0.05). The correlation between dependent variables was also determined using Pearson's correlation.Principal component analysis (PCA) was applied to explore patterns and potential correlations among variables. Statistical analyses were performed using SPSS for Windows Version 26.0 (IBM corporation, New York, USA).

## 3 Results and discussion

### 3.1 Physicochemical properties

#### 3.1.1 Moisture content (MC)

**Fig. 2:**
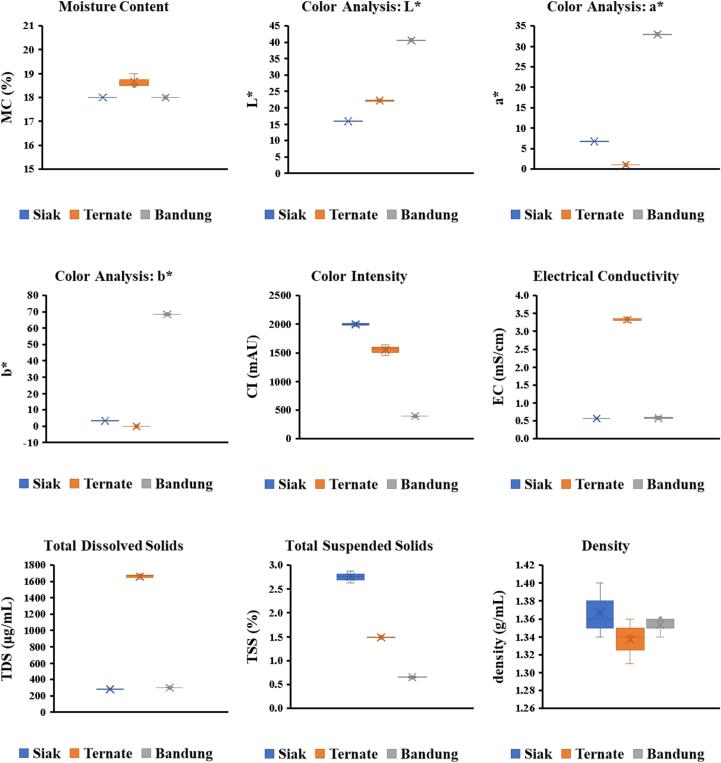
Physical properties of Indonesian *Apis dorsata* honey.

The MC of *A. dorsata* honey samples ranged from 18.00 % to 18.67 % (Fig. 2). These findings align with Belay et al. (2013), who reported MC values between 17.29 % and 18.49 % for Harenna forest honey (Ethiopia). The results also showed lower MC compared to other Indonesian *A. dorsata* honey, ranging from 19.14 % to 29.20 % (Riswahyuli et al., 2020). Honey's hygroscopic properties, particularly fructose's high water solubility, facilitate moisture absorption from the air (Buba et al., 2013). Forested areas typically exhibit high humidity, potentially increasing MC. However, the analyzed forest honey displayed relatively low moisture levels. The observed MC values fall below the maximum permissible thresholds of 20 % (Codex Alimentarius) and 22 % (Standar Nasional Indonesia). The relatively low MC may be attributed to intact beeswax layers in honeycombs and optimal harvesting and postharvest handling practices. MC significantly influences honey's stability and quality. The study findings reveal similarities among three samples, potentially influenced by similar geographical conditions (temperature, relative humidity, precipitation). Although this study's limited sample size restricts broader generalizations, the results provide valuable insights into MC characteristics of *A. dorsata* honey from Indonesia's tropical forests.

#### 3.1.2 Color analysis

Color plays a crucial role in honey acceptance, influencing consumer preferences (Kek et al., 2018; Scholz et al., 2020). González-Miret et al. (2005) classified honey colors based on lightness (L*) values. Our results (Fig. 2) indicate dark-toned colors for all samples, with L* values below 50 (Rababah et al., 2014). Sample Siak exhibited the darkest color (L* = 15.96 ± 0.07), while sample Bandung showed the brightest (L* = 40.58 ± 0.14). Positive a* values placed all samples in the red color range. Samples Siak and Bandung displayed positive b* values (yellow range), whereas sample Ternate showed a negative b* value (blue hue). The dark color likely results from high pigment, polyphenol, and mineral content (K, Ca, Na, Mg, Cu, Fe, Zn, Ni, Al, Mn, Cd) derived from nectar sources (Bertoncelj et al., 2007). Variations in honey color may also stem from Maillard reaction, producing melanoidin (Riswahyuli et al., 2020). Forest-origin honey's association with mineral-rich soils likely contributes to the observed dark colors.

#### 3.1.3 Color intensity (Abs_450_)

Honey color intensity can be used to indicate the presence of pigments with antioxidant activity like flavonoids and carotenoids, contributing to the total antioxidant capacity (Khalil et al., 2012; Moniruzzaman et al., 2013). The results showed that Siak honey had the highest color intensity (1997.67 ± 32.50 mAU), while Bandung honey had the lowest (394.00 ± 4.00 mAU). Comparative studies reported color intensity values of 475.5 ± 82.4 mAU for Tualang honey and 446.5 ± 153.2 mAU for Gelam honey from Malaysia (Kek et al., 2018), and 544.33 ± 11.68 mAU for Tualang honey (Moniruzzaman et al., 2013). Variations in color intensity likely stem from nectar sources, geographical origins, and environmental factors influencing chemical composition (Bertoncelj et al., 2007). Postharvest handling, such as excessive heating or prolonged storage, may also impact color intensity through caramelization or Maillard reaction. Although color intensity contributes to antioxidant properties, our correlation test and PCA (Table 2; Fig. 5) did not reveal a strong relationship, likely due to multiple influencing factors.

**Table 2:** Bivariate Pearson's Correlation between variables on Indonesian *Apis dorsata* Honey.

Correlations (r value)																					
Variables	MC	L⁎	a⁎	b⁎	CI	EC	TDS	TSS	Dnst	DN	HMF	pH	TA	Ash	Pro	RS	TPC	TFC	IC_50_	AEAC	AAE
MC	1	−0.242	−0.586	−0.493	0.205	0.922⁎⁎	0.922⁎⁎	−0.108	−0.416	0.903⁎⁎	0.844⁎⁎	0.690⁎	0.863⁎⁎	0.935⁎⁎	0.261	−0.901⁎⁎	0.914⁎⁎	0.902⁎⁎	−0.785⁎	0.882⁎⁎	0.906⁎⁎
L*		1	0.914⁎⁎	0.957⁎⁎	−0.998⁎⁎	−0.266	−0.260	−0.921⁎⁎	−0.101	−0.415	−0.626	0.423	−0.584	−0.268	0.849⁎⁎	0.053	−0.170	−0.411	−0.262	0.000	−0.321
a*			1	0.992⁎⁎	−0.902⁎⁎	−0.635	−0.630	−0.687⁎	0.123	−0.749⁎	−0.889⁎⁎	0.019	−0.863⁎⁎	−0.636	0.563	0.452	−0.555	−0.746⁎	0.152	−0.406	−0.676⁎
b*				1	−0.948⁎⁎	−0.534	−0.528	−0.772⁎	0.057	−0.660	−0.824⁎⁎	0.143	−0.793⁎	−0.535	0.661	0.338	−0.447	−0.657	0.028	−0.288	−0.580
CI					1	0.244	0.237	0.928⁎⁎	0.097	0.394	0.606	−0.442	0.563	0.243	−0.858⁎⁎	−0.027	0.146	0.390	0.284	−0.023	0.298
EC						1	1.000⁎⁎	−0.123	−0.491	0.987⁎⁎	0.918⁎⁎	0.760⁎	0.936⁎⁎	0.999⁎⁎	0.278	−0.972⁎⁎	0.995⁎⁎	0.987⁎⁎	−0.859⁎⁎	0.962⁎⁎	0.995⁎⁎
TDS							1	−0.130	−0.490	0.986⁎⁎	0.916⁎⁎	0.764⁎	0.934⁎⁎	0.999⁎⁎	0.283	−0.973⁎⁎	0.995⁎⁎	0.986⁎⁎	−0.862⁎⁎	0.964⁎⁎	0.994⁎⁎
TSS								1	0.336	0.034	0.278	−0.737⁎	0.228	−0.121	−0.981⁎⁎	0.337	−0.221	0.029	0.609	−0.382	−0.065
Dnst									1	−0.436	−0.348	−0.522	−0.359	−0.474	−0.383	0.556	−0.503	−0.437	0.515	−0.528	−0.422
DN										1	0.969⁎⁎	0.648	0.978⁎⁎	0.986⁎⁎	0.122	−0.926⁎⁎	0.967⁎⁎	1.000⁎⁎	−0.768⁎	0.909⁎⁎	0.992⁎⁎
HMF											1	0.442	0.998⁎⁎	0.918⁎⁎	−0.125	−0.808⁎⁎	0.875⁎⁎	0.968⁎⁎	−0.588	0.779⁎	0.938⁎⁎
pH												1	0.488	0.757⁎	0.830⁎⁎	−0.877⁎⁎	0.821⁎⁎	0.652	−0.983⁎⁎	0.904⁎⁎	0.721⁎
TA													1	0.937⁎⁎	−0.075	−0.837⁎⁎	0.897⁎⁎	0.979⁎⁎	−0.627	0.807⁎⁎	0.955⁎⁎
Ash														1	0.275	−0.971⁎⁎	0.994⁎⁎	0.987⁎⁎	−0.857⁎⁎	0.961⁎⁎	0.994⁎⁎
Pro															1	−0.471	0.370	0.125	−0.724⁎	0.522	0.217
RS																1	−0.989⁎⁎	−0.927⁎⁎	0.944⁎⁎	−0.993⁎⁎	−0.954⁎⁎
TPC																	1	0.968⁎⁎	−0.905⁎⁎	0.984⁎⁎	0.985⁎⁎
TFC																		1	−0.770⁎	0.910⁎⁎	0.994⁎⁎
IC_50_																			1	−0.964⁎⁎	−0.826⁎⁎
AEAC																				1	0.942⁎⁎
AAE																					1

⁎ Correlation is significant at the 0.05 level (2-tailed).

⁎⁎ Correlation is significant at the 0.01 level (2-tailed).

#### 3.1.4 Electrical conductivity (EC) and total dissolved solids (TDS)

EC measures the ability of organic and inorganic substances to ionize and conduct electricity (Yücel & Sultanoğlu, 2013). This study found EC values ranging from 0.56 to 3.32 mS/cm (Fig. 2). Siak and Bandung honey met Codex Alimentarius standards (<0.8 mS/cm) (2001), aligning with Rysha et al.'s (2022) findings for European honeys (0.07–0.60 mS/cm). The correlation test and PCA (Fig. 5) confirmed relationships between EC values, total acidity, and ash content. Geographical and ecological variations influenced floral composition, contributing to differences in EC values. Notably, Ternate honey exceeded regulatory limits. EC and TDS values reflect mineral content variations. TDS measures organic and inorganic materials (Moniruzzaman et al., 2013). Our TDS values ranged from 283.00 to 1660.00 μg/mL (Fig. 2). Siak and Bandung honey showed similar TDS values, while Ternate honey exhibited significantly higher values. Comparative studies reported TDS values of 54–260 μg/mL for Bangladeshi honey (Trisha et al., 2023) and 138–1506 μg/mL for Malaysian honey (Moniruzzaman et al., 2014). Higher TDS values likely result from increased organic and inorganic substances, particularly minerals like potassium and sodium.

#### 3.1.5 Total suspended solids (TSS)

The TSS quantify insoluble impurities in honey, including organic and inorganic substances. This study found TSS values ranging from 0.65 % (Bandung honey) to 2.76 % (Siak honey) (Fig. 2). These values exceed Codex Alimentarius and Standar Nasional Indonesia standards (<0.5 %). Comparatively, Ahmida et al. (2013) reported lower TSS values (0.029–0.073 %) for Libyan honey, indicating fewer impurities. Elevated TSS values in *A. dorsata* honey likely result from poor hygienic practices during harvesting, extraction methods (pressing, centrifugation, filtration), and suboptimal post-harvest handling (Desfita et al., 2022). The excessive TSS values suggest contamination during processing. Regulatory bodies should consider common post-harvest practices when reviewing standards for raw honey. This study's findings highlight concerns for honey farmers and regulatory agencies regarding post-harvest processing and standards alignment. The limited sample size, restricts broader generalizations.

#### 3.1.6 Density

This study found minimal variations in density among Siak, Ternate, and Bandung honeys, ranging from 1.34 to 1.37 g/mL (Fig. 2). These values align with established standards (Codex Alimentarius, France) and previous research (Amir et al., 2010; Khalil et al., 2012), indicating consistency across regions and floral sources. Honey density primarily depends on moisture content; higher density corresponds to lower moisture levels. Elevated moisture triggers fermentation, reducing density as sugars break down. PCA results confirm negative correlations between density and moisture content, and positive correlations with reducing sugars (Table 2; Fig. 5).

#### 3.1.7 Diastase activity

**Fig. 3:**
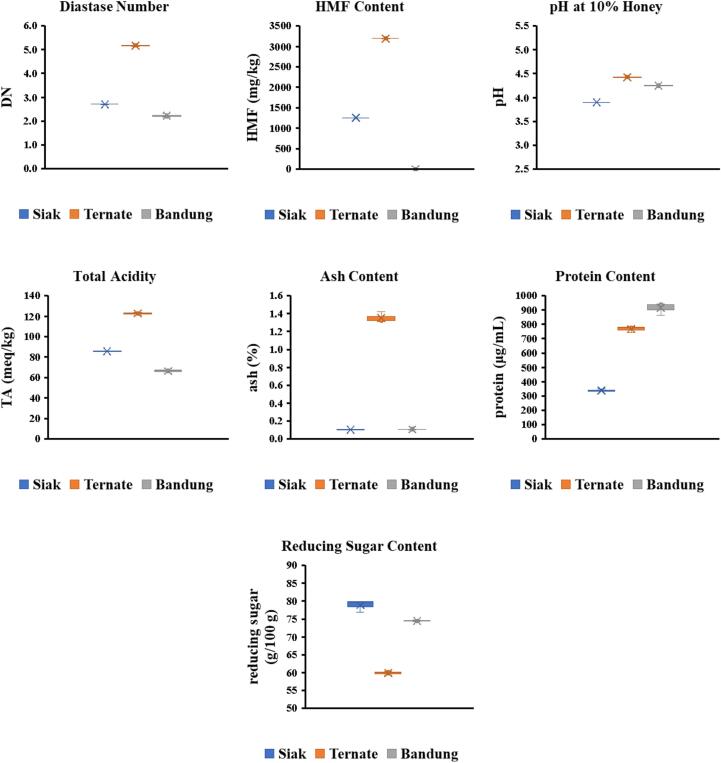
Chemical properties of Indonesian *Apis dorsata* honey.

Diastase activity, a key indicator of honey freshness, varied among *A. dorsata* honey samples, ranging from 2.23 DN (Bandung) to 5.17 DN (Ternate) (Fig. 3). These values are significantly lower compared to studies by Buba et al. (2013) and Nayik et al. (2015), reporting diastase activities of 8.00–13.00 DN and 12.18–22.52 DN for Nigerian and Indian honey, respectively. According to Standar Nasional Indonesia, the minimum prescribed value of diastase activity in forest honey is 1 DN, which all three samples met. However, none met the Codex Alimentarius standard of 8 DN. The variations in diastase activity among Siak, Ternate, and Bandung honey could be attributed to factors such as differences in nectar type, heat treatment, storage conditions, and storage duration. Additionally, bee species may also contribute to variations in diastase activity (Desfita et al., 2022). Our findings across all samples indicate that diastase activity did not meet the limits established by the Codex Alimentarius regulation. Given the naturally low diastase activity in tropical forest honey, we recommend that regulatory bodies reassess or issue a policy brief accommodating pure, unadulterated honey. This would ensure standards align with practical conditions in tropical regions, particularly Indonesia. This study's limited sample size warrants further research.

#### 3.1.8 Hydroxymethylfurfural (HMF) content

HMF, a compound formed from sugar decomposition through the Maillard reaction, assesses honey freshness. Fresh honey typically contains less than 1 mg/kg of HMF (Damto et al., 2023). This study found HMF levels ranging from 0.36 mg/kg (Bandung) to 3193.10 mg/kg (Ternate) in *A. dorsata* honey samples (Fig. 3). Bandung honey had significantly lower HMF content compared to Siak (1257.75 mg/kg) and Ternate honeys. According to Codex Alimentarius and Standar Nasional Indonesia standards, maximum HMF content is 80 mg/kg and 40 mg/kg, respectively. Only Bandung honey met these standards. Our findings exceeded those reported for Riau Province, Indonesia (0.49–27.84 mg/kg) and Serbia (1.19–3.37 mg/kg) honeys (Desfita et al., 2022; Sakač et al., 2019). Elevated HMF levels may result from factors like prolonged storage, aging, heat treatment, acidity, moisture content, sugar composition, amino acid, and mineral content (Buba et al., 2013; Gela et al., 2021). Our study highlights the need for regulatory bodies to reassess HMF limits, considering processing methods and natural factors influencing forest honey from tropical regions like Indonesia. These preliminary results necessitate additional investigations with expanded sample sizes to confirm and generalize the observations.

#### 3.1.9 pH value and Total acidity (TA)

Honey's natural acidity, typically ranging the pH from 3.5 to 5.5 (Bogdanov et al., 1997), contributes to its stability. *A. dorsata* honey samples exhibited pH values between 3.90 (Siak) and 4.43 (Ternate) (Fig. 3). Siak honey had the lowest pH, followed by Bandung (4.25) and Ternate. These results align with Algerian honey (3.64–4.59) (Bouhala et al., 2020). The acidity primarily stems from organic acids, particularly gluconic acid, produced during honey maturation (Karabagias et al., 2014). Total acidity (TA) levels varied significantly, ranging from 66.25 meq/kg (Bandung) to 122.57 meq/kg (Ternate) (Fig. 3). These values surpass Codex Alimentarius and Standar Nasional Indonesia standards (50 meq/kg). Our findings exceed Greek honey (23.75–44.94 meq/kg) (Karabagias et al., 2014) but align with Sudanese *A. florea* honey (98.40 meq/kg) (Al-Ghamdi et al., 2019). High acidity may result from factors like nectar acid content, honey fermentation by osmotolerant yeasts (Gomes et al., 2010), or natural variations. Moisture and sugar content, within established standards except for Ternate honey, suggest natural acidity fluctuations. Our study highlights the need for regulatory bodies to reassess standards, accommodating pure, unadulterated honey from tropical regions like Indonesia, where natural acidity levels may exceed current limits. Further comprehensive research with larger sample size is necessary to validate these findings.

#### 3.1.10 Ash content

The ash content of honey, reflecting inorganic compounds and mineral concentrations, serves as a quality indicator revealing purity and environmental contamination (Bouhala et al., 2020). Ash content influences sensory properties like color, taste and aroma. Higher ash levels often result in darker honey with altered flavor profiles. *A. dorsata* honey samples exhibited ash content ranging from 0.10 % (Siak) to 1.35 % (Ternate) (Fig. 3). Siak and Bandung honey met Standar Nasional Indonesia's maximum limit (0.5 %), whereas Ternate honey exceeded this standard. Similar findings were reported for *A. florea* honey from Sudan (1.16 %) (Al-Ghamdi et al., 2019). The elevated ash content may be attributed to mineral content, including K, Ca, Mg, Zn and Fe (Sakač et al., 2019). Geographic location, climate and soil conditions influence floral sources and mineral content, contributing to variations in ash content (Desfita et al., 2022).

#### 3.1.11 Protein content

Honey typically contains small amounts of protein, less than 5000 μg/mL, primarily consisting of enzymes like diastase, invertase, glucose oxidase and catalase (Saxena et al., 2010). The determination of protein content was conducted using BSA-standard curve with BSA concentration ranging from 0 to 1000 μg/mL. The curve has a linear equation of Y = 0.0009X + 0.0145 with R^2^ = 0.9987. Protein content of honey samples exhibited varying protein content: Siak (338.89 μg/mL), Ternate (768.52 μg/mL) and Bandung (915.19 μg/mL) (Fig. 3). These findings align with Brazilian honey (121–1121 μg/mL) (Liberato et al., 2013). However, Indian (480–2293 μg/mL) and Malaysian (2040–4830 μg/mL) honey reported higher protein content (Moniruzzaman et al., 2013; Saxena et al., 2010). Protein variations across honey samples may be attributed to environmental factors, geographical origin and floral sources, influencing nectar's protein composition (Moniruzzaman et al., 2013).

#### 3.1.12 Reducing sugar content (RS)

Glucose and fructose, honey's primary monosaccharides, comprise approximately 70–80 % of its reducing sugar content (RS). Reducing sugar levels closely relate to moisture content and acidity, as fermentation affects these parameters. High moisture content promotes osmotolerant yeast activity, converting sugars into carbon dioxide, alcohol and acetic acid, reducing sugar content and increasing acidity. The reducing sugars was determined using a standard curve of glucose with concentrations ranging from 0.1 to 0.6 mg/mL. The curve equation was Y = 1.1364X − 0.0982 with R^2^ = 0.9935. The reducing sugar content of *A. dorsata* honey ranged from 59.88 g/100 g (Ternate) to 78.90 g/100 g (Siak) (Fig. 3). Ternate honey had significantly lower reducing sugar content, followed by Bandung (74.54 g/100 g) and Siak (78.90 g/100 g). Except for Ternate honey, all samples met Standar Nasional Indonesia (65 g/100 g) and Codex Alimentarius standards (60 g/100 g). These findings align with Indian honey (71.43–75.92 g/100 g) (Nayik et al., 2015) but differ from Indian honey studied by Saxena et al. (2010), which reported lower reducing sugar content (43.3–58.1 g/100 g). Lower reducing sugar content may result from factors like fermentation, overheating, prolonged storage or conversion of sugars to HMF (Saxena et al., 2010). Geographical location, botanical origin, harvest timing, processing methods and storage conditions also influence reducing sugar variations. Further comprehensive research with larger sample sizes is necessary to validate these results and provide more definitive conclusions regarding reducing sugar content in *A. dorsata* honey.

### 3.2 Antioxidant properties

#### 3.2.1 Total Phenolic Content (TPC)

**Fig. 4:**
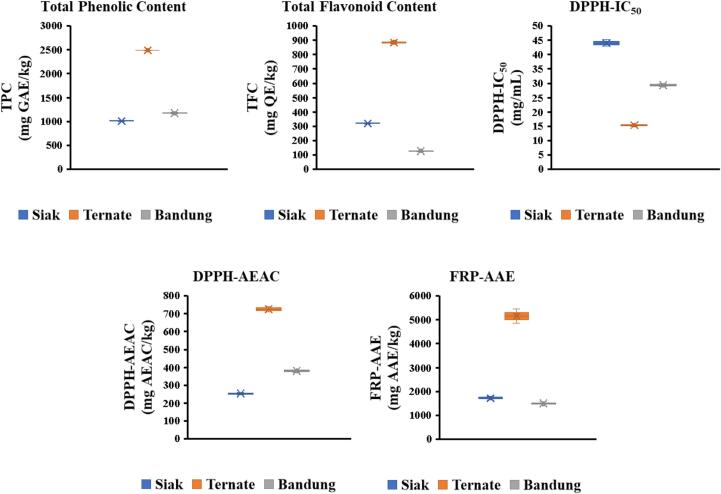
Antioxidant properties of Indonesian *Apis dorsata* honey.

The TPC was calculated using a gallic acid standard curve (0–200 μg/mL), yielding the eq. Y = 0.0043 + 0.0126, with R^2^ = 0.9967. The TPC of Indonesian *A. dorsata* honey samples from Siak (Riau), Ternate (North Maluku), and Bandung (West Java) exhibited significant regional variations. Ternate honey had the highest TPC (2488.89 ± 5.72 mg GAE/kg), while Siak honey showed the lowest TPC (1010.75 ± 13.42 mg GAE/kg) (Fig. 4). These findings indicate that Indonesian *A. dorsata* honey has notably higher phenolic content compared to Malaysian Gelam honey (78.43 mg GAE/100 g) (Kek et al., 2018) and comparable levels to Tualang honey (251.7 mg GAE/kg honey) (Mohamed et al., 2010). Variations in TPC levels among the samples likely stem from botanical sources, regional differences in bioactive compounds and floral diversity.

Phenolic compounds, including flavonoids and phenolic acids, play a crucial role in neutralizing free radicals and reducing oxidative stress. The high TPC in Ternate honey may be attributed to diverse floral species contributing a broader range of phenolic compounds. These bioactive compounds provide antioxidant activity, anti-inflammatory and antimicrobial benefits, enhancing the value of forest honey as a functional food (Ávila et al., 2019; Borsato et al., 2014; Mduda et al., 2023; Saputra & Nurlina, 2022). Future studies with expanded sample sizes will provide deeper insights into regional variations and potential therapeutic benefits.

#### 3.2.2 Total Flavonoid Content (TFC)

The TFC analysis revealed similar regional trends among Indonesian *A. dorsata* honey samples. TPC determination employed a quercetin calibration curve (0–100 μg/mL, yielding a strong linear correlation (R^2^ = 0.9995), with eq. Y = 0.0066X − 0.0138. Ternate honey exhibited the highest TFC (885.49 ± 9.32 mg QE/kg), whereas Bandung honey showed the lowest (126.89 ± 4.24 mg QE/kg) (Fig. 4). These findings align with previous research indicating floral origin, geographical location and environmental conditions influence flavonoid levels (Kaligis & Mokosuli, 2022). Although lower than Manembo forest honey (1.6 mg QE/g), the flavonoid levels in this study demonstrate significant antioxidant contributions. Flavonoids play a crucial role in antioxidant defense by neutralizing free radicals and chelating metal ions. The higher TFC in Ternate honey suggests potential benefits in combating oxidative stress and promoting cellular health. The synergistic interplay between TPC and TFC enhances honey's overall antioxidant capacity, positioning it as a potent natural antioxidant source.

#### 3.2.3 Antioxidant activity by DPPH assay

The antioxidant activity of Indonesian *A. dorsata* honey samples, assessed via DPPH assay, revealed significant regional variations. Ternate honey exhibited the highest antioxidant activity with the lowest IC_50_ value (15.37 ± 0.32 mg/mL), while Siak honey showed the lowest antioxidant activity (IC_50_ = 44.06 ± 1.19 mg/mL). Morover, the AEAC value also calculated using an ascorbic acid calibration curve (0–14 μg/mL) with eq. Y = 4.5255X - 0.5306 and R^2^ = 0.9954. The AEAC values further confirmed these findings, with Ternate honey recording the highest value (726.22 ± 15.19 mg AEAC/kg), significantly exceeding Siak honey (253.54 ± 6.84 mg AEAC/kg). Compared to Tualang honey from Malaysia, which recorded an AEAC of 244.10 mg AEAC/kg (Moniruzzaman et al., 2013), the Indonesian *A. dorsata* honey, particularly from Ternate, demonstrated superior antioxidant potential. These differences in antioxidant capacity could be attributed to the presence of bioactive compounds such as polyphenols, carotenoids, tocopherols and ascorbic acid. The lower IC_50_ of honey from Ternate also reflects the higher efficiency of antioxidants in neutralizing free radicals, showcasing its potential as a functional ingredient in nutraceutical applications. The interplay between antioxidant components likely contributes to the robust scavenging activity observed in this sample.

#### 3.2.4 Antioxidant activity by FRP assay

The FRP assay further confirmed the high antioxidant activity of Indonesian *A. dorsata* honey. The ascorbic acid standard curve (0–100 μg/mL) enabled calculation of antioxidant activity equivalent (AAE) which represent the FRP, with a linear regression equation: Y = 0.0081X + 0.00009 (R^2^ = 0.9995). Ternate honey exhibited the highest AAE value (5154.75 ± 298.70 mg AAE/kg), followed by Siak (1724.73 ± 73.27 mg AAE/kg) and Bandung (1509.86 ± 51.15 mg AAE/kg) (Fig. 4). These findings surpass Malaysian Tualang honey (233.4 mg AAE/kg) (Kek et al., 2018), highlighting regional floral sources and environmental factor's impact on honey's bioactive profile. The FRAP assay demonstrates antioxidant's ability to reduce Fe^3+^ to Fe^2+^, preventing harmful hydroxyl radical formation. Indonesian *A. dorsata* honey's high AAE values suggest robust ferric-reducing abilities, likely linked to rich phenolic and flavonoid content. Combined TPC, TFC, DPPH and FRAP assay results underscore Indonesian *A. dorsata* honey's significant antioxidant potential. Future studies with larger sample sizes are necessary to confirm these findings and explore potential geographical and seasonal variations.

### 3.3 Pearson's Correlation

The Pearson's correlation analysis (Table 2) revealed significant relationships among the studied variables. Color parameters (L*, a*, b*, and color intensity) exhibited strong intercorrelations (*r* > 0.7), indicating interdependence. MC, EC, TDS, DN, HMF, TA, ash, and RS showed strong correlations (r > 0.7), suggesting shared physicochemical processes. Notably, TSS were strongly influenced by L* and CI, highlighting the interplay between physical properties and suspended components. Chemical properties, such as HMF and TA, showed strong correlations with color parameters (a*, b*), implying a relationship between chemical composition and honey's appearance. Ash content exhibited strong correlations with MC, EC, TDS, DN, HMF, and TA. Protein content (Pro) correlated strongly with L*, CI, TSS, and pH. Reducing sugar content exhibited a strong correlation with pH. The weak correlation between pH and TA (*r* = 0.488) may be attributed to the presence of weak organic acids (Kek et al., 2018).

Antioxidant characteristics, including TPC, TFC, IC_50_ values, and antioxidant capacity (AEAC and AAE), demonstrated significant relationships with various physicochemical properties. TPC correlated with moisture, EC, TDS, DN, HMF, pH, TA, ash content, and RS. TFC showed significant relationships with moisture, a*, EC, TDS, DN, HMF, TA, ash content, RS, TPC. IC_50_ values were strongly associated with moisture, EC, TDS, DN, pH, ash, RS, TPC, and TFC. Antioxidant capacity (AEAC and AAE) were influenced by nearly all measured variables, including phenolic and flavonoid contents.

These findings emphasize the interconnections between antioxidant properties and physical/chemical profiles, providing valuable insights into Indonesian *A. dorsata* honey's characteristics. Future studies with larger sample sizes will enhance the reliability and generalizability of these correlations, informing potential applications in functional food development.

### 3.4 Principal component analysis

**Fig. 5:**
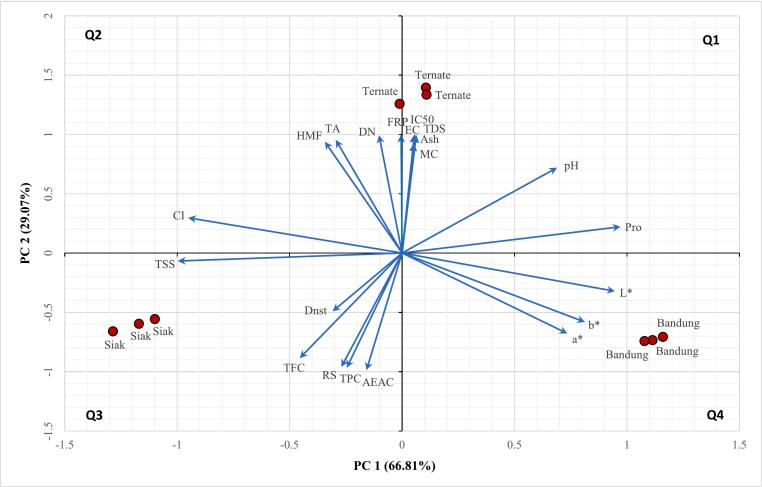
Biplot graphics of physicochemical and antioxidant properties of Indonesian *Apis dorsata* honey.

The PCA was conducted to examine the data and illustrate the relationships between the samples and their physicochemical and antioxidant properties (Fig. 5). This analysis produced a two-factor model, which accounted for 95.88 % of the total variance in the data. The variables included in the PCA were MC, color values L*, a*, and b*, CI, EC, TDS, TSS, density (Dnst), DN, HMF, pH, TA, ash, Pro, RS, TPC, TFC, antioxidant activity by DPPH (IC_50_ and AEAC), and antioxidant activity by FRP. The first principle component (PC1) explained 66.81 % of the total variance, with MC, EC, TDS, DN, HMF, pH, TA, Ash, IC_50_, and FRP being the most influential variables on this component. The second principle component (PC2), which accounted for 29.07 % of the total variance, was primarily associated with L*, a*, b*, pH, and Pro.

The score plot and loading plot of PC1 and PC2, accounting for 95.88 % of the data variance, revealed the relationships between the physicochemical and antioxidant properties of the honey samples and their geographical origins (Fig. 5.). The relative positions of the points in the plot indicated statistical differences in these properties among the samples. In the score plot, closely positioned sample points indicate similarity in characteristics, whereas distantly positioned points suggest significant differences between the samples. In the loading plot, if the vectors between variables forming small angles, the variables indicate a positive correlation. Conversely, if the vectors forming a large angle approaching 180 degrees, the variables exhibit a negative correlation. If two vectors form a 90-degree angle, there is no correlation between the two variables. In the overall biplot, sample points (dots) located near variable points (vectors) indicate that the samples have high values for the corresponding variables.

The biplot graphics showed that Siak honey was distinct in quadrant 3 (Q3), showing high values in proximity to variables such as CI, TSS, Dnst, and RS. Ternate honey, also distincted in quadrant 1 (Q1), exhibiting high values for MC, EC, TDS, DN, HMF, pH, TA, and Ash. Bandung honey was also distincted in quadrant 4, showing high values for L*, a*, b*, and Pro. These findings validate that PCA, based on physicochemical and antioxidant data, effectively differentiates three samples of Indonesian *A. dorsata* honey according to its geographical origin. The resulting biplot from PCA also showed a positive correlation between MC, EC, TDS, DN, HMF, pH, TA, Ash, IC50, and FRP. Another positive correlation was also found between parameter L*, a*, b*, and Pro. The negative correlation can be found between CI and L*, AEAC and FRP, AEAC and EC, etc. Variables such as CI and RS, as well as L* and RS, showed no correlation between them.

## 4 Conclusion

This study highlights the significant variability in the physicochemical and antioxidant properties of *A. dorsata* honey collected from three regions in Indonesia, reflecting the influence of the country's vast tropical forests and diverse nectar sources. Physicochemical analysis reveals that the honey samples in present sudy meet international standards (Codex Alimentarius) for MC, density, and RS. Notably, Siak honey complies with EC standards, while Bandung honey meets both EC and HMF standards. However, TSS, DN, and TA exceed recommended limits. These findings are expected to be a part for providing implications for the regulatory framework, highlighting the need to incorporate characteristics of tropical honey, such as Indonesian varieties, into standardization guidelines. Ternate honey exhibited the highest antioxidant activities, while Siak honey had the highest reducing sugar content, highlighting the impact of geographical origins and local floral sources. Key physicochemical properties strongly correlated with antioxidant characteristics, including MC, EC, TDS, DN, HMF, pH, TA, ash, and RS. These properties highlight critical parameters for understanding and enhancing honey's antioxidant potential.

While this study provides valuable insights, its limitations due to small sample size should be acknowledged. Future studies involving larger, diverse samples (considering seasonal and regional variations) are essential for deriving robust conclusions and supporting potential updates to regulatory standards. Expanding analysis to additional parameters (such as sucrose, fructose, glucose, and carbon-4 plant sugars), as well as investigating antimicrobial properties and metabolomic profiling would enrich characterization of *A. dorsata* honey. Such efforts would complement the present findings, advancing knowledge of Indonesian forest honey and highlighting its distinctive qualities and potential applications.

## Ethical approval

Not applicable.

## Concent to participate

Not applicable.

## Concent to publish

All authors read and approved the publishing of this article.

## CRediT authorship contribution statement

**Mahani:** Writing – original draft, Supervision, Resources, Methodology, Funding acquisition, Conceptualization. **Pamungkas Rizki Ferdian:** Writing – original draft, Supervision, Methodology, Funding acquisition, Formal analysis, Conceptualization. **Habil Muhammad Ghibran:** Writing – original draft, Formal analysis, Data curation. **Amirah Fathia Herlina:** Writing – original draft, Formal analysis, Data curation. **Siti Nurhasanah:** Writing – review & editing, Supervision, Methodology, Formal analysis. **Nunung Nurjanah:** Writing – review & editing, Methodology, Investigation, Formal analysis. **Rizki Rabeca Elfirta:** Writing – review & editing, Methodology, Formal analysis. **Avry Pribadi:** Writing – review & editing, Resources, Formal analysis. **Raden Lia Rahadian Amalia:** Writing – review & editing, Project administration, Methodology, Data curation. **I Made Samudra:** Writing – review & editing, Supervision, Formal analysis.

## Declaration of competing interest

The authors declare that they have no known competing financial interest or personal relationship that could have appeared to influence the work reported in this paper.

## Data Availability

Data will be made available on request.
